# Serum Vitamin D Deficiency and Male Infertility: A Relationship?

**DOI:** 10.7759/cureus.56070

**Published:** 2024-03-13

**Authors:** Opeyemi R Akinajo, Gbenga Olorunfemi, Philip O Oshun, Moses A Ogunjimi, Ayodeji A Oluwole

**Affiliations:** 1 Department of Obstetrics and Gynecology, Lagos University Teaching Hospital, Lagos, NGA; 2 Division of Epidemiology and Biostatistics, School of Public Health, University of the Witwatersrand, Johannesburg, ZAF; 3 Department of Medical Microbiology and Parasitology, College of Medicine, University of Lagos, Lagos, NGA; 4 Department of Surgery, Division of Urology, Lagos University Teaching Hospital, Lagos, NGA; 5 Department of Obstetrics and Gynecology, College of Medicine, University of Lagos, Lagos, NGA

**Keywords:** semen analysis, semen parameters, vitamin d sufficiency, vitamin d insufficiency, vitamin d deficiency, male infertility, infertility

## Abstract

Background

Male infertility is one of the major reproductive health concerns, causing a lot of distress for couples globally. Others have looked into its connection to vitamin D deficiency, but their findings are conflicting.

Aim

This study aimed to determine the relationship between male infertility and vitamin D deficiency among Nigerians.

Method

This analytical cross-sectional study was conducted among 132 men. A purposive sampling technique was employed to recruit 66 participants in the study (men with infertility) and control groups (men with proven fertility). Descriptive statistics were conducted, while the association between vitamin D level and sperm parameters was assessed using bivariate and regression modeling. A two-tailed test of the hypothesis was assumed, and the level of statistical significance was set at a P-value < 0.05.

Results

None of the participants had a serum vitamin D deficiency. However, the overall serum vitamin D insufficiency rate was 15%. The median vitamin D level for the total study population (both fertile and infertile) was 37.52 ng/ml (IQR: 32.1 - 51.69). This study demonstrated no association between serum vitamin D levels and male infertility, as well as no association between serum vitamin D levels and the quality of semen parameters.

Conclusion

There was no significant association between vitamin D levels, male infertility, and seminal fluid parameters. However, larger multi-center studies are recommended to provide further insights into this conclusion.

## Introduction

Globally, infertility is one of the major reproductive health concerns for couples, often neglected, and it is frequently viewed as mainly the female partner's problem, especially in resource-limited settings [1]. This erroneous assumption is fraught with the risk of stigmatization, neglect, distress, depression, and discrimination among the affected females [1]. Infertility is a global problem with a varied prevalence rate ranging from one region to another, with an estimated impact on 60 to 80 million couples globally [2]. In Nigeria, the rate of infertility among couples ranges between 20 and 30% [3]. About 31% [4] and 38% [5] of the male partners had abnormal semen quality, with 15-30% of couples diagnosed with unexplained infertility [6].

It was postulated that vitamin D deficiency may cause poor-quality sperm production [7]. Vitamin D deficiency is a well-recognized, significant public health problem associated with many conditions, including male and female infertility [7]. The role of vitamin D in reproduction and fertility through its receptors in reproductive tissues has been reported [8]. However, these reports vary widely, generating controversy [9]. 

A literature search in our environment and sub-region yielded a dearth of information on vitamin D deficiency, which is amenable to treatment. Hence, evaluating its relationship with infertility in our environment is necessary. Therefore, this study aimed to compare the serum vitamin D levels between fertile and infertile men and assess the effects of vitamin D levels on semen parameters.

## Materials and methods

Study design

This study was an analytical prospective cross-sectional comparative study conducted at the gynecology and urology outpatient clinics of the Lagos University Teaching Hospital (LUTH), Idi Araba, Lagos, Nigeria, from December 2017 to December 2018. This hospital is one of the three tertiary institutions located in the Lagos metropolis, acting as a tertiary referral center for hospitals in both the public and private sectors of Lagos and neighboring states.

Study participants

These were male partners of women who presented at the gynecology outpatient clinic and men who presented at the urology outpatient clinic with a 12-month history of infertility. The infertile groups were men with abnormal semen parameters whose partners had been evaluated and diagnosed as fertile. This group is not limited to men who have never fathered a child but also involves men who have had a previous history of fathering a child or more, whether in their present or previous relationships, but could not achieve conception for at least 12 months. In contrast, the comparative group were men with normal semen parameters whose partner is being managed for infertility secondary to female factors.

In all, 66 fertile and 66 infertile men were recruited. The total sample size for the study was determined based on the formula for comparison of proportions, n = (Zα/2 + Zβ)2 P1(1-P1)/(Po-P1)2, and a 20% attrition. The power of the study was assumed to be 80% with a 95% confidence interval.

Sampling technique and recruitment strategy

We approached eligible participants purposively at their respective outpatient clinics. A clinical evaluation was done for each participant at the initial visit, and a tentative diagnosis was made. We excluded men with a significant history of systemic or chronic diseases, a history of intake of supplements containing vitamin D or anabolic steroids, previous groin surgery (e.g., vasectomy, hydrocelectomy), and the presence of any malignancy. Men with normal semen parameters previously managed for infertility were also excluded.

We provided eligible participants with detailed information about the importance of the study, procedures, and their right to voluntary participation. Those who voluntarily gave informed consent were enrolled consecutively. All participants abstained from sexual intercourse for three to five days, and semen production was masturbation. Samples were collected into sterile universal containers and submitted for seminal fluid analysis (SFA) at the hospital's main laboratory within an hour of production at body temperature (37°C).

Data collection techniques 

Data were collected following approval from the institutional ethical committee. An interviewer-administered questionnaire was used to elicit information from the participants. The first section elicited information on the socio-demographic characteristics of the participants: age, educational status, religion, occupation, marital status, family setting, type of infertility, duration of infertility, etc. The second section elicited information on the results of their semen analysis and serum vitamin D status.

Laboratory method for semen analysis

Following semen sample collection, analysis was performed by a skilled laboratory scientist at the main hospital laboratory according to the methods and standards outlined by WHO, 2010 [10]. The parameters assessed for the expected typical values include a volume of 1.5 ml or more. Results were presented as normospermia (sperm concentration of > 15 x 106 cells/ml), oligospermia (sperm count below 15 million/ml), azoospermia (absence of spermatozoa in the ejaculate), asthenospermia (reduced sperm motility of < 40%), and teratospermia (reduced sperm morphology of < 4% of all normal form).

Blood sample collection and analysis

Using the median cubital vein, 5 ml of blood was drawn from each participant between 8 a.m. and 10 a.m. after eight to 12 hours of overnight fasting and immediately transported to the laboratory in serum ethylenediaminetetraacetic acid (EDTA) specimen bottles for analysis. The test principle for the vitamin D assay is based on competitive enzyme-linked immunoassays (ELISA). Following strict analytical procedures by a skilled laboratory scientist, which were done according to the kit's instructions, the results were interpreted as vitamin D deficiency: < 10 ng/ml, insufficiency: 10-30 ng/ml, sufficiency: 30-100 ng/ml, and intoxication: > 100 ng/ml (the definition was based on the reference range of the ELISA kit used in this study).

Data analysis

Data entry, coding, cleaning, and analysis were done using STATA, version 16.0 (StataCorp. 2019. Stata Statistical Software: Release 16. College Station, TX: StataCorp LLC). Descriptive statistics such as means and medians were used to summarize quantitative variables, while categorical variables were described using proportions and percentages. Bivariate analysis was used to investigate the association between variables and the outcome of interest. Univariable and multivariable linear and logistic regression were conducted to further assess the association between vitamin D and sperm parameters after correcting for confounding variables. A two-tailed test of the hypothesis was assumed, and the level of statistical significance was set at a P-value < 0.05.

## Results

Socio-demographic status of participants

A total of 132 men participated in this study, with 66 categorized as men with infertility (case) and fertile men (control), respectively. The mean age was 40.43 ± 6.65 years, and all 66 participants were married (100%). The majority of the participants were of the Yoruba ethnic group (n = 75, 56.82%), Christians (n = 94, 71.21%), and attained tertiary educational level (n = 72, 54.55%). Most participants were traders or businessmen (n = 62, 46.97%), followed by artisans (n = 20, 15.15%). There is, however, no statistically significant difference found (Table 1).

**Table 1:** Comparison of socio-biological parameters among fertile and infertile participants #: Student’s independent t-test; $: Fischer’s exact test; ^: Mann Whitney U test; €: Pearson’s Chi-square

Characteristics	Infertile (Case) N= 66 (%)	Fertile (Control) N= 66 (%)	Total	Test statistics	P-value
Age (mean ± SD), Years	39.85±6.38	41.02 ±6.92	40.43 ± 6.65	-1.107	0.3156^#^
<30	2 (3.03)	1(1.52)	3 (2.27)		0.759^$^
30- 39	31(46.97)	27(40.91)	58 (43.94)		
40-49	28 (42.42)	31(46.97)	59(44.70)		
≥50	5(7.58)	7 (10.94)	12 (9.09)		
Number of children (Median, IQR)	0(0-0)	0(0-1)	0(0-5)	-1.745	0.0809^
0	54 (81.82)	45 (68.18)	99 (75.00)	3.3486	0.187^$^
1-2	8 (12.12)	15 (20.73)	23 (17.42)		
≥3	4 (6.06)	6 (9.09)	10 (7.58)		
Tribe					
Yoruba	42 (63.64)	33 (50.00)	75 (56.82)	3.3486	0.187^€^
Igbo	19 (28.79)	21 (31.82)	40 (30.30)		
Others	5 (7.58)	12 (18.18)	17 (12.88)		
Religion					
Christianity	46 (69.70)	48 (72.73)	94(71.21)	4.0624	0.131^€^
Islam	20 (30.30)	18 (27.27)	38(28.79)		
Educational qualification					
Primary	3(4.55)	3 (4.55)	6 (4.55)		0.587^$^
Secondary	18 (27.27)	19 (28.79)	37(28.03)		
Tertiary	39(59.09)	33(50.00)	72 (54.55)		
Postgraduate	6(9.09)	11 (16.67)	17 (12.88)		
Occupation					
Student	2 (3.03)	0(0.00)	2 (1.55)		0.140^$^
Artisan	14 (21.21)	6 (9.09)	20(15.15)		
Trader/Businessman	31 (46.97)	31 (46.97)	62 (46.97)		
Civil servant	8 (12.12)	11 (16.67)	19 (14.39)		
Health worker	1 (1.52)	4 (6.06)	5 (3.79)		
Others	6 (9.09)	10 (15.15)	16 (12.12)		

Infertility history of participants

The duration of infertility among respondents in each group ranged from one to 21 years, with a mean of 5.92 ± 5.17 (case) and 5.08 ± 4.33 (control), respectively. The majority of men (n = 62, 93.94%) with infertility had the primary type.

Serum vitamin D levels among infertile and fertile men

None of the participants had serum vitamin D deficiency; however, the overall rate of vitamin D insufficiency was 15.91%, with no statistically significant difference (Table 2). After utilizing the median vitamin D level of the study population (37.52 ng/ml (IQR: 32.136 - 51.692)), a cut-off for normal vitamin D levels was categorized into low (< 37.52) and high (≥ 37.524) vitamin D levels, with no statistically significant difference (Table 2).

**Table 2:** Serum vitamin D levels across the infertile and fertile groups ^: The median vitamin D level was utilized to categorize into low and normal values Units of vitamin D levels are in ng/ml

Characteristics	Infertile (Case) N = 66(%)	Fertile (Control) mean N = 66(%)	Total	Test statistics	P-value
Vitamin D levels (mean, SD)					
	41.43±12.40	42.20±14.03	41.81±13.20	-0.3356	0.7377^#^
Vitamin D category					
Deficient	0 (0.0)	0(0.0)	0(0.0)		0.475^$^
Insufficient	9 (13.64)	12 (18.18)	21 (15.91)		
Sufficient	57 (86.36)	54 (81.82)	111 (84.09)		
Vitamin D category by median value ^					
Low (<37.5245)	33	33	66	1.00	
High (≥37.5245)	33	33	66		

Semen parameters among fertile and infertile men

The distribution of semen parameters in the case group includes 21 cases of azoospermia (31.82%), 38 of oligospermia (57.58%), 7 of normospermia (10.61%), 47 of asthenospermia (71.21%), 19 of normal motility (28.79%), 22 of teratospermia (33.33%), and 44 of normal morphology (66.67%). Those with multiple sperm abnormalities had oligoasthenospermia (n = 19, 28.79%) and teratoasthenospermia (n = 1, 1.52%). There was a statistically significant difference between the semen parameters among the infertile and fertile men (P-value < 0.0001). The comparison of semen parameters using a 95% confidence interval also yielded statistically significant differences between the study groups for sperm count, morphology, and motility (P-value < 0.0001).

Association between vitamin D levels and semen parameters

**Figure 1:**
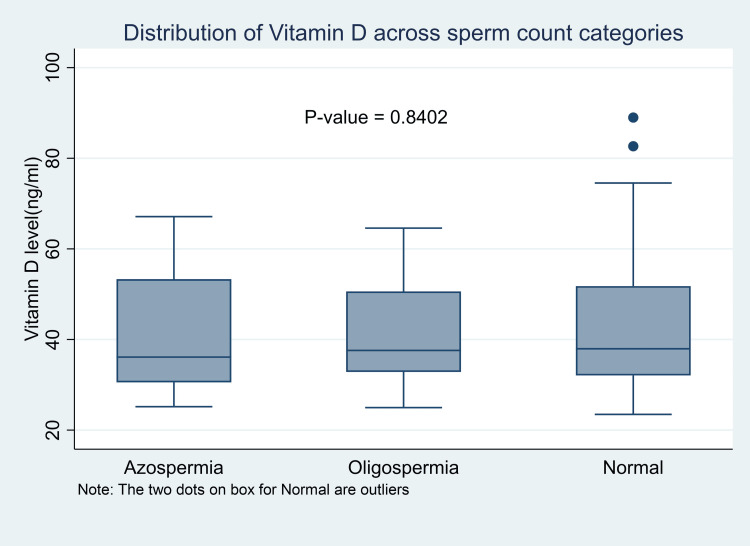
Association between median VD levels and sperm parameters among fertile and infertile men

The association between vitamin D levels and semen parameters yielded no statistically significant relationship (Table 3). Neither was there any statistically significant difference in the median vitamin D levels among participants with normal and abnormal sperm counts (normal sperm count vs. oligospermia vs. azoospermia: 37.95 ng/ml (32.06 - 51.78) vs. 37.59 ng/ml (32.83 - 50.61) vs. 36.11 ng/ml (30.54 - 53.32), P-value = 0.9265) (Figure 1). 

**Table 3:** Association between vitamin D levels and sperm parameters among all participants

Characteristics	Insufficient vitamin D levels	Sufficient vitamin D level	P-value
Sperm count categories			
Azoospermia (0/ejaculate)	5 (23.81)	16 (14.41)	0.403
Oligospermia (<15 x 10^6^)	4 (19.05)	34(30.63)	
Normospermia (≥ 15 x 10^6^)	12 (57.14)	61(54.95)	
Motility			
Asthenozoospermia (<40% motile)	7 (33.33)	40 (36.04)	0.813
Normal (≥40% motile)	14 (66.67)	71(63.96)	
Morphology			
Teratozoospermia (< 4% normal morphology)	5 (23.81)	17 (15.32)	0.338
Normal (≥ 4% normal form)	16 (76.19)	94(84.68)	
WHO classification of normal /abnormal semen*			
Abnormal	9(42.86)	57(51.35)	0.475
Normal	12(57.14)	54(48.65)	

Correlation between vitamin D and sperm parameters

There was no statistically significant correlation between sperm counts and vitamin D levels among fertile and infertile men.

## Discussion

This comparative study examined the possible association between vitamin D levels, sperm parameters, and male infertility in our environment. We investigated the serum concentration of vitamin D levels among fertile and infertile Nigerian men to determine the level and the association of its deficiency with their semen parameters. However, this study did not reveal any vitamin D deficiency or association between low or normal vitamin D levels and male infertility.

The mean age of participants in the studied population was 40.43 ± 6.65 years, which was in keeping with the mean age of 41 ± 7.3 years and 43.72 ± 1.5 years obtained from some previous studies on infertility [11,12] but more than the 33.5 ± 4.8 years, 30.3 ± 5.7 years, and 29.24 years reported from other studies [13-15]. The duration of infertility in our study ranged from one to 21 years, with a mean of 5.92 ± 5.17 years for the cases and 5.08 ± 4.33 years for the control, respectively. This result is close to the mean of 6.9 ± 5.4 years revealed in a cross-sectional study [11], but more than 4.26 ± 3.61 years and 2 ± 3 years in some other observational studies [11,16,17]. The rate of primary infertility among participants in the current study was 93.94%, which was way above the rate of 67% observed in the northern part of Nigeria [18]. This finding was, however, in contrast with various Nigerian studies where secondary infertility was found to be the main type of infertility among their participants [4,5,11,19]. In those studies, previous surgeries, such as herniorrhaphy, varicocelectomy, and hydrocelectomy, were some of the most common factors associated with secondary infertility. These factors were some of the exclusion criteria in our study, which could explain the high rate of primary infertility among our participants.

It is of note to state that different values have been used to define vitamin D deficiency over the past years, as evidenced by the guidelines developed by the Endocrine Society (ES) and the Institute of Medicine (IOM), where desired cut-off levels were set at 30 ng/ml and 20 ng/ml, respectively [20,21]. Even though they were widely adopted, these values were selected based on studies conducted in high-income countries [7]. Therefore, due to a lack of consensus on a definitive reference value for optimal serum vitamin D levels and no defined established value from studies conducted in our environment, vitamin D deficiency in our study was described as a value less than 10 ng/mL based on our study's ELISA kit reference range (the Calbiotech Kit (catalog number: VD220B)). And with this value, our study revealed no vitamin D deficiency among participants. Given this, we used the median vitamin D level from our study populace as a cut-off value for low and high vitamin D levels (low < 37 ng/mL and high > 37 ng/mL). However, our findings still revealed no statistically significant association between these levels and male infertility.

This absence of vitamin D deficiency could be due to adequate and regular exposure to sunlight by the cohort of young and active men in our study while going through their daily duties (most of the participants were traders or businessmen, followed by artisans). It has been estimated that twice-weekly sun exposure between 10 a.m. and 3 p.m. for at least five to 30 minutes is adequate for maintaining sufficient vitamin D levels [7]. Most men in our study would have exceeded this minimum estimated sun exposure based on the nature of their occupation. However, compared to the observations in a study in Poland, where a similar vitamin D level to ours was used as a cut-off for vitamin D deficiency (<10 ng/ml), the prevalence among their study populace was nearly 40% [22]. However, in contrast to our study, their high prevalence rate was attributed to a lack of adequate sunlight exposure among a large proportion of their participants [22].

Even though there was no vitamin D deficiency among the participants, our findings revealed that less than one-third (15%) of the study populace had insufficient serum vitamin D levels, giving a vitamin D sufficient rate of 84%. This finding is very similar to the observed rates from a cross-sectional study where deficiency, insufficiency, and sufficiency among the fertile group were 1 (0.5%), 17 (9%), and 168 (90%), respectively, while 4 (3%), 36 (28%), and 87 (68%) were found in the altered sperm parameter group, respectively [23]. In contrast to these findings, most participants in other studies were vitamin D deficient, and only a small percentage had normal vitamin D levels [22,24]. These variations have been attributed to different factors, including geographical locations and inadequate sun exposure [22,24].

Surprisingly, findings from our study revealed a higher rate of vitamin D insufficiency among fertile men when compared to infertile men. However, there is no statistically significant association between this vitamin D level and any semen parameters among fertile men. Similar to our findings, some observational studies revealed no association between sperm parameters and vitamin D levels in normospermic men [13,22]. However, contrary to these observations, a cross-sectional study found a positive association between high and low vitamin D levels and semen parameters among young, healthy men, suggesting a possible inverted 'U-shaped' relation [25]. According to the findings in that study, this positive correlation might be because of the effect of vitamin D on intracellular calcium and spermatogenesis, with the possibility that a low level could reduce this effect. In contrast, a higher level could alter vitamin D's systemic and local concentration, hence its positive correlation with male infertility [25]. They, however, advised further studies to identify the cause of the association between these vitamin D levels and male infertility.

Even though the insufficient vitamin D level among infertile men in our study was low, we could not find any significant association between it and poor semen quality. A report from a retrospective study revealed no correlations between serum vitamin D levels and total motility, progressive motility, count, volume, or sperm morphology [26]. Similarly, in a pilot study, neither dietary intake of foods rich in vitamin D nor serum vitamin D were found to be associated with semen parameters or reproductive hormone levels [27]. There were also no substantial differences in sperm concentration, sperm progressive motility, or normal form among men with sufficient and insufficient vitamin D groups [27]. Explanations for this lack of association may include the apparent limitations of current routine semen analysis, which does not check for sperm dysfunction, thus making it an imperfect predictor of male fertility [27]. Furthermore, vitamin D's lack of direct effect on spermatogenesis may also be responsible for the observations [23].

Findings from our study revealed that men with abnormalities in all categories of semen parameters had sufficient serum vitamin D levels, and this was similar to the results from Hammoud et al.'s study [25], which also yielded no statistically significant association in any of the categories between serum vitamin D levels and any of the types of semen parameters in their study.

Overall, the role of vitamin D in semen quality from various studies remains inconsistent and may result from the apparent variability in the definitions of normal and abnormal serum vitamin D levels. These inconsistencies may have resulted from the different cut-off values for serum vitamin D levels used in these studies [28,29]. The methodology of the vitamin D assay may be another reason for the variation in the observations from these studies. The most widely used assay is liquid chromatography with tandem mass spectrometry (LC-MS/MS) [28,30]. Another popular method is chemiluminescent immunoassay (CLIA) [25,27]. In our study, serum vitamin D levels were determined by the ELISA method with the Calbiotech Kit.

The main strength of our study is that it's a single-centered design study with a single laboratory facility, which reduces the possibility of inter-laboratory variation. Additionally, the samples (semen and blood samples) were handled and analyzed by a skilled laboratory scientist in semen and vitamin D analysis, respectively, with reduced risk of inter-individual variations, which could compromise the results and, hence, the study's outcome. 

However, the recruited participants may not accurately represent the general population because this was a hospital-based study. Though the sample size was achieved with a high completion rate, the number of participants was still relatively small. A larger sample size might have demonstrated a significant association between vitamin D levels and semen parameters among men with infertility and fertile men.

Furthermore, there is a risk of the inability to counteract the effect of intra-individual variability of sperm parameters with single semen sample collection; however, it has been shown that a single ejaculate can sufficiently predict the overall semen quality [11,12]. In our study, we employed a single semen sample collection from all participants due to time factors to reduce the cost implication (all semen and blood samples were analyzed at no cost to all participants) and to reduce the risk of less participation from refusal to produce a second semen sample.

## Conclusions

Our study aimed to investigate the association between serum vitamin D deficiency and male infertility by comparing fertile and infertile Nigerian men. The results showed that there was no vitamin D deficiency among the participants. However, 15% of the total population had vitamin D insufficiency, which did not show any association with male infertility or semen parameters. Therefore, it can be concluded that low vitamin D levels are not the likely risk factor for the poor semen quality observed in this group of men. To gain further insight, future research with a larger sample size, including both fertile and infertile male groups, is needed, considering seasonal variations in serum vitamin D levels.
